# Supplementary material for: Comprehensive Characterization of the Coding and Non-Coding Single Nucleotide Polymorphisms in the Tumor Protein p63 (TP63) Gene Using In Silico Tools
Source: Biomolecules. 2021 Nov 20;11(11):1733. doi: 10.3390/biom11111733 (PMC8637305; doi:10.3390/biom11111733)
Supplement: Supplementary file 1 [file biomolecules-11-01733-s001.zip › biomolecules-1441232-supplementary.pdf]

Supplementary Tables and Figures

Comprehensive Characterization of the Coding and Non-coding Single Nucleotide Polymorphisms in the Tumor Protein p63 (TP63) Gene Using In Silico Tools

Akter. Shamima[1], Hossain. Shafaat[2], Ali. Md Ackas [3], Hosen. Md. Ismail[2], Shekhar. Hossain Uddin[2*]

1. Department of Bioinformatics and Computational Biology, George Mason University, Fairfax, Virginia- 22030, USA
2. Clinical Biochemistry and Translational Medicine Laboratory, Department of Biochemistry and Molecular Biology, University of Dhaka, Dhaka-1000, Bangladesh.
3. Division of Computer Aided Drug-Design, The Red-Green Research Center, 16, Tejkunipara, Tejgaon, Dhaka-1215, Bangladesh

* Correspondence to: Email: hossainshekhar@du.ac.bd

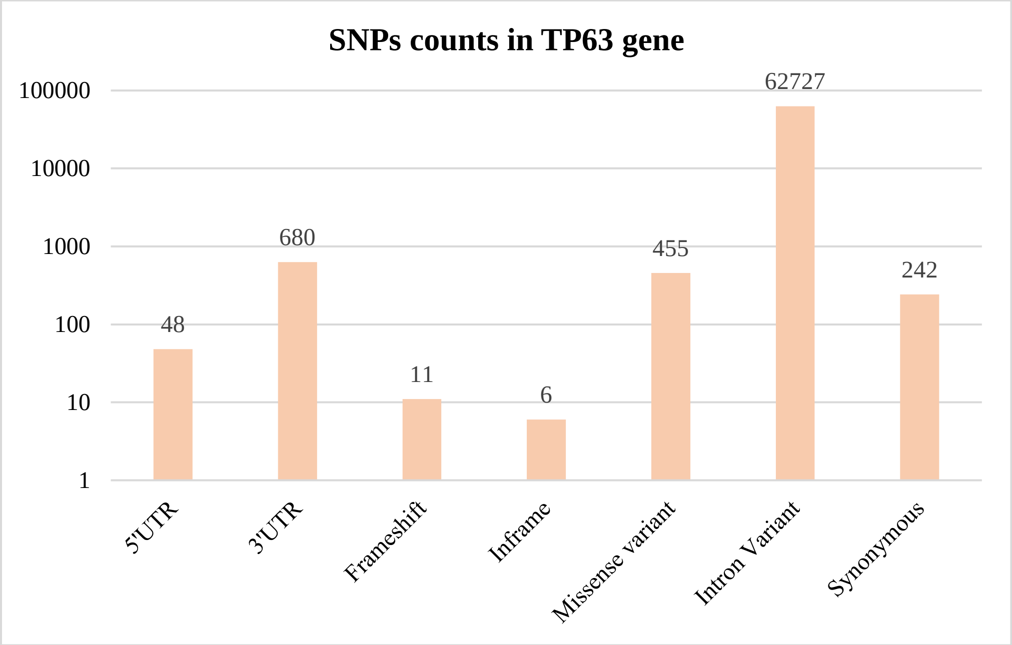

Figure S1. SNP types and number of TP63

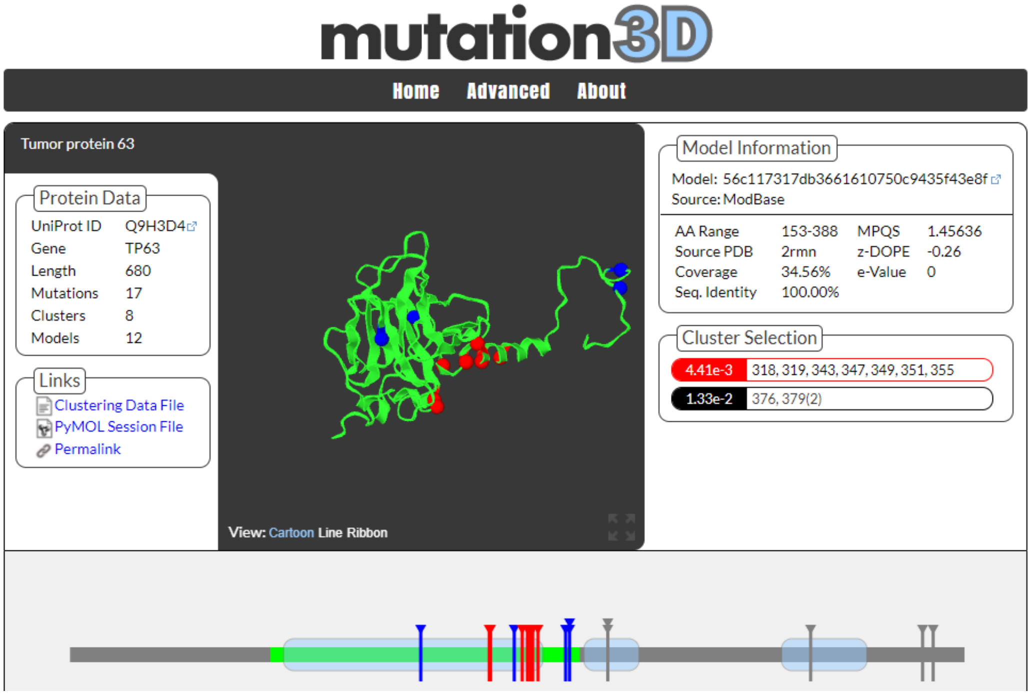

Figure S2. Mutations in protein structure. The positions of the SNPs are R266Q, R318H, R319H, R343Q, R337Q, C347F, D355N, G349E, R376C, R408C, R408H, R376C, R379C, R379H, L562R, R647H, R655Q. *(This figure has been downloaded from Mutation 3D website after analysis; Mutation3D* http://mutation3d.org)

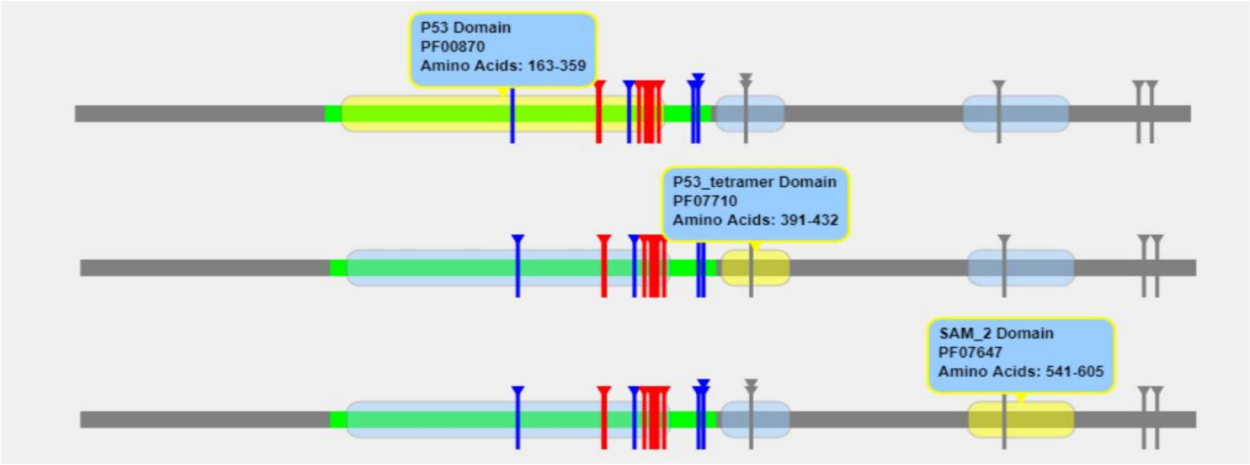

Figure S3. Three domains are shown in yellow color with blue box labeling after structural analysis with Mutation 3D. Vertical sticks show the mutation in different domains. P53 domain is the DNA binding domain of TP63. R266Q, R318H, R319H, R337Q, R343Q, C347F, D351G, G349E, D355N, are present in p53 domain or DNA binding domain, R376C, R408C, R408H, R379C, R379H are in p53_tetramer domain and L562R in Sam domain.

A wild typeR319

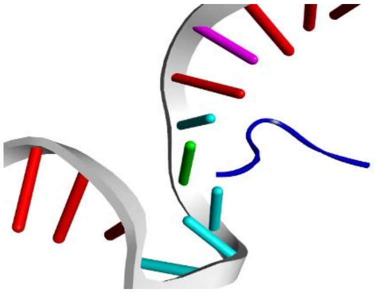

B wild type R319

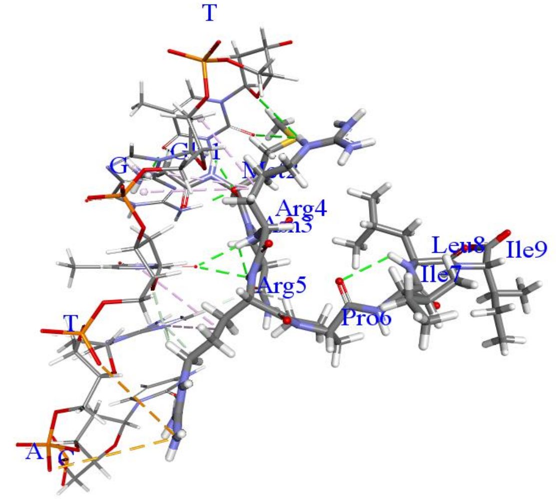

C Mutant H319

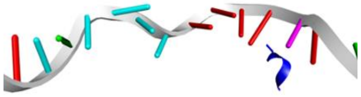

D Mutant H319

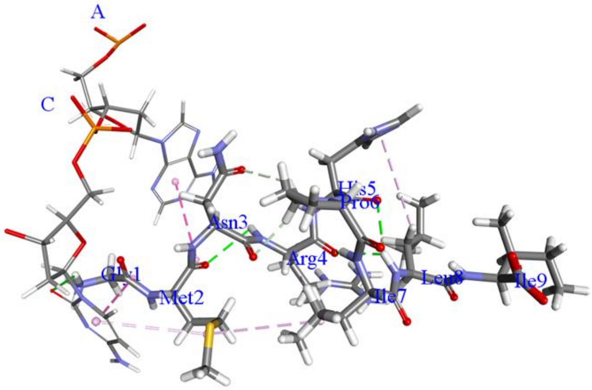

E Wild type G349

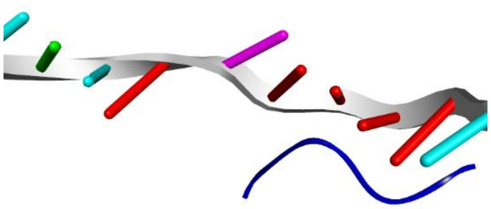

F wild type G349

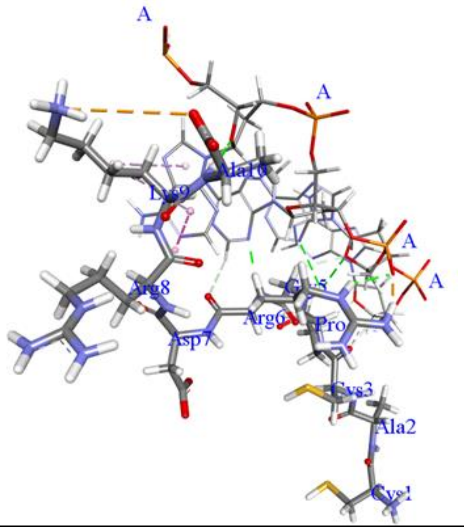

G Mutant E349

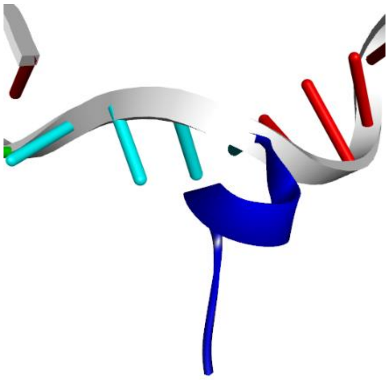

H Mutant E349

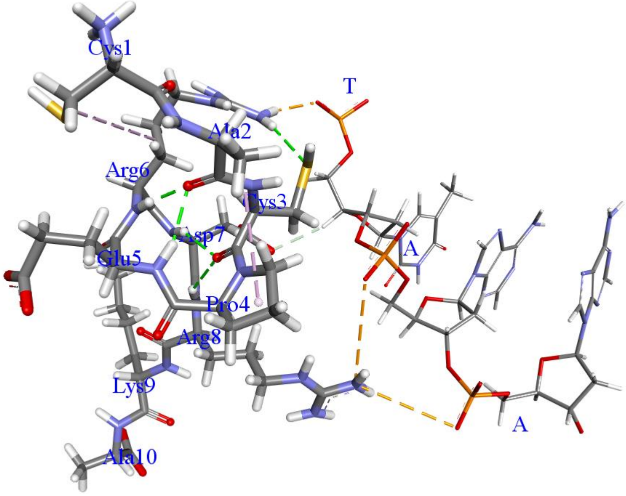

I Wild type 347

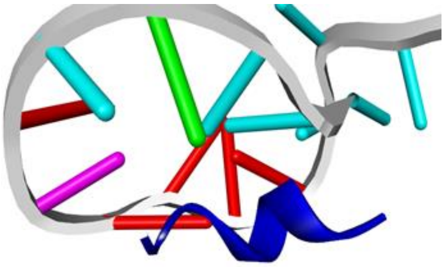

J wild type C347

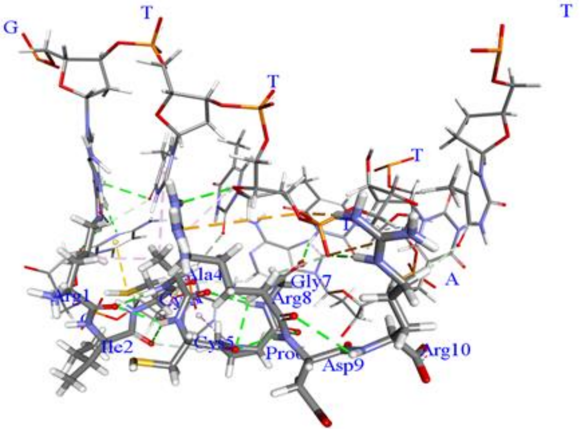

K Mutant 347

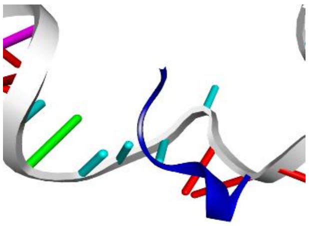

L Mutant F347

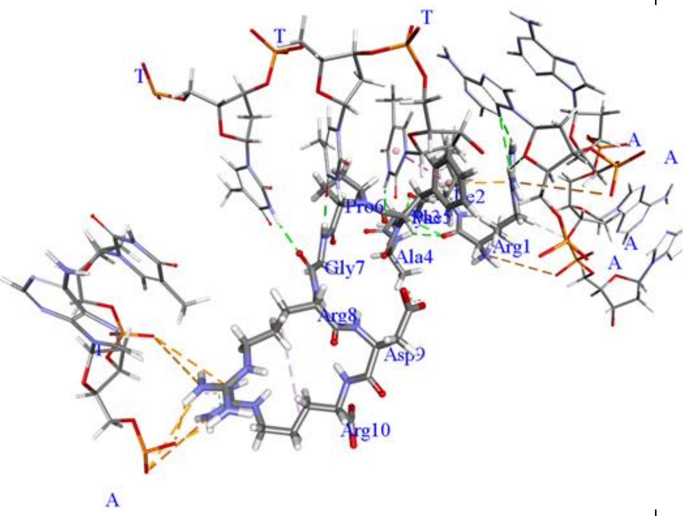

Figure S4. Non-bonding interactions of wild type TP63 and mutant TP63 proteins at 319, 349, and 347 positions (A,B,C,D,E,F,G,H,I,J,K,L) generated from the 250ns snapshot of MD simulation.

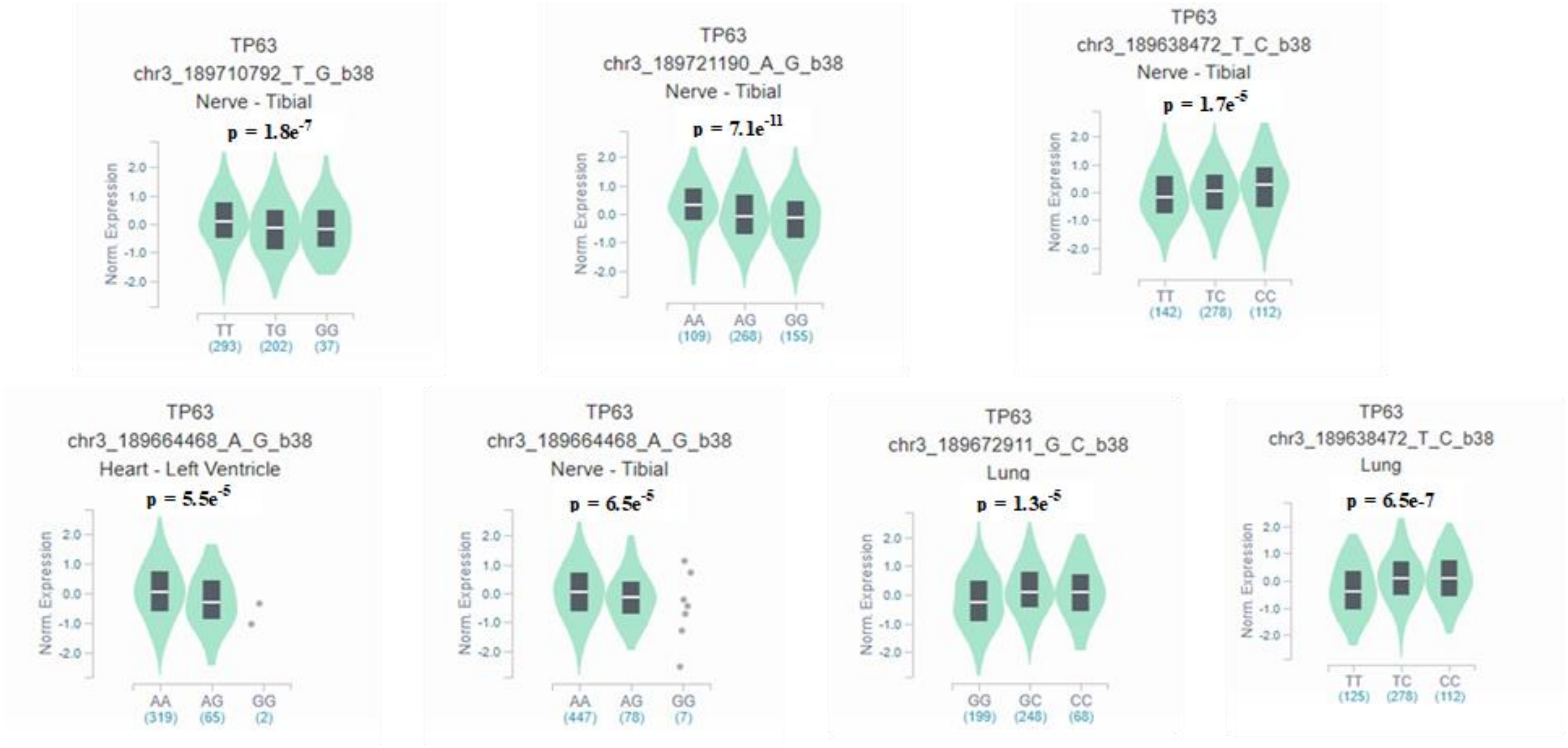

Figure S5. Violin plots of noncoding SNPs for single tissue eQTLs through analyzing with GTEx portal. The plots show the normalized TP63 gene expressions with mutations in different tissues along with significant p values.

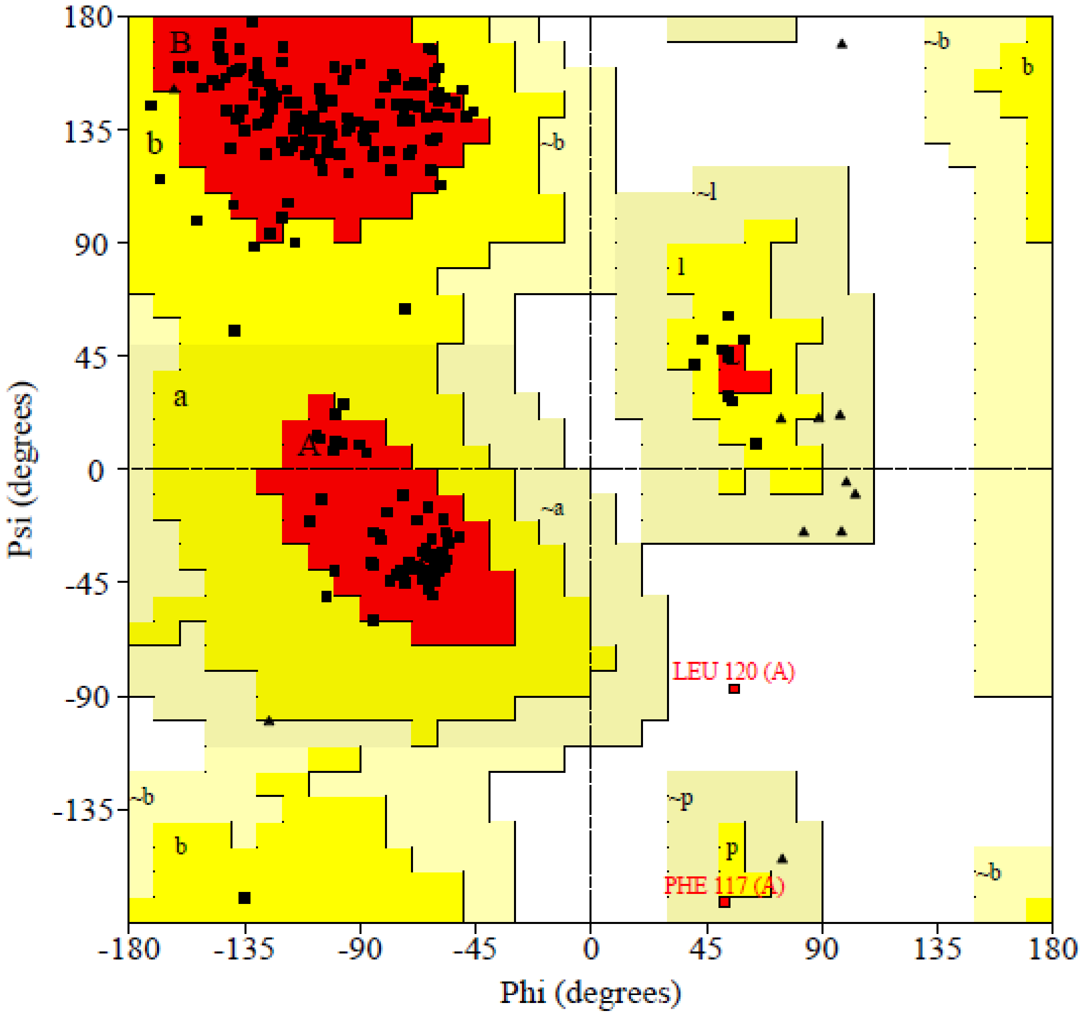

Plot statistics

Residues in most favoured regions [A,B,L]	173	86.9%
Residues in additional allowed regions [a,b,l,p]	24	12.1%
Residues in generously allowed regions [~a,~b,~l,~p]	1	0.5%
Residues in disallowed regions	1	0.5%
	----	------
Number of non-glycine and non-proline residues	199	100.0%
Number of end-residues (excl. Gly and Pro)	1	
Number of glycine residues (shown as triangles)	14	
Number of proline residues	19	

Total number of residues	233	

Figure S6: Ramachandran plot statistics of Precheck analysis in PDB sum server for 3D structure of TP63 protein: 2RMN.A,B,L denote the alpha, beta, and loop structures in protein.

Table S1. Functional nsSNPs Prediction in TP63 in SIFT, PolyPhen2, CADD

Variant ID	SNP	Source	Conseq.Type (SNP type)	SIFT_class	PolyPhen2 class	CADD_class
rs1266601767	D178Y	dbSNP	missense variant	deleterious	probably damaging	likely deleterious
rs866938979	S189L	dbSNP	missense variant	deleterious	probably damaging	likely deleterious
rs1057517984	Y202C	dbSNP	missense variant	deleterious	probably damaging	likely deleterious
rs121908849	R266Q	dbSNP	missense variant	deleterious	probably damaging	likely deleterious
rs121908840	R318H	dbSNP	missense variant	deleterious	probably damaging	likely deleterious
rs886039442	R319H	dbSNP	missense variant	deleterious	probably damaging	likely deleterious
rs1320920860	V325D	dbSNP	missense variant	deleterious	probably damaging	likely deleterious
rs753404887	D331V	dbSNP	missense variant	deleterious	probably damaging	likely deleterious
rs1040062725	G332R	dbSNP	missense variant	deleterious	probably damaging	likely deleterious
rs113993967	R337Q	dbSNP	missense variant	deleterious	probably damaging	likely deleterious
rs1029852196	R338H	dbSNP	missense variant	deleterious	probably damaging	likely deleterious
rs121908841	R343Q	dbSNP	missense variant	deleterious	probably damaging	likely deleterious
rs1064793282	C347F	dbSNP	missense variant	deleterious	probably damaging	likely deleterious
rs866267914	G349E	dbSNP	missense variant	deleterious	probably damaging	likely deleterious
rs121908844	D351G	dbSNP	missense variant	deleterious	probably damaging	likely deleterious
rs1553857889	D355N	dbSNP	missense variant	deleterious	probably damaging	likely deleterious
rs757536818	R376C	dbSNP	missense variant	deleterious	probably damaging	likely deleterious
rs761885185	R379C	dbSNP	missense variant	deleterious	probably damaging	likely deleterious
rs765502786	R379H	dbSNP	missense variant	deleterious	probably damaging	likely deleterious
rs1173679499	R393Q	dbSNP	missense variant	deleterious	probably damaging	likely deleterious
rs1282887680	R408C	dbSNP	missense variant	deleterious	probably damaging	likely deleterious
rs751698974	R408H	dbSNP	missense variant	deleterious	probably damaging	likely deleterious
rs886039443	F552C	dbSNP	missense variant	deleterious	probably damaging	likely deleterious
rs121908843	C561G	dbSNP	missense variant	deleterious	probably damaging	likely deleterious
rs774221257	L562R	dbSNP	missense variant	deleterious	probably damaging	likely deleterious
rs1172845743	Y574C	dbSNP	missense variant	deleterious	probably damaging	likely deleterious
rs774550896	R647H	dbSNP	missense variant	deleterious	probably damaging	likely deleterious
rs764601563	R655Q	dbSNP	missense variant	deleterious	probably damaging	likely deleterious

Table S2. Analysis of nsSNPs using PROVEAN and ClinVar

Variant ID	SNP	Source	PROVEAN score	PROVEAN impact	ClinVar result
rs1266601767	D178Y	dbSNP	-3.381	Deleterious	not found
rs866938979	S189L	dbSNP	-5.556	Deleterious	not found
rs1057517984	Y202C	dbSNP	-7.964	Deleterious	Likely pathogenic
rs121908849	R266Q	dbSNP	-3.612	Deleterious	Pathogenic
rs121908840	R318H	dbSNP	-4.645	Deleterious	Pathogenic
rs886039442	R319H	dbSNP	-4.627	Deleterious	Pathogenic
rs1320920860	V325D	dbSNP	-4.628	Deleterious	not found
rs753404887	D331V	dbSNP	-6.694	Deleterious	not found
rs1040062725	G332R	dbSNP	-5.704	Deleterious	not found
rs113993967	R337Q	dbSNP	-3.618	Deleterious	Pathogenic
rs1029852196	R338H	dbSNP	-4.523	Deleterious	Uncertain significance
rs121908841	R343Q	dbSNP	-3.663	Deleterious	Pathogenic
rs1064793282	C347F	dbSNP	-10.073	Deleterious	Pathogenic
rs866267914	G349E	dbSNP	-7.342	Deleterious	Pathogenic
rs121908844	D351G	dbSNP	-6.41	Deleterious	Pathogenic
rs1553857889	D355N	dbSNP	-3.512	Deleterious	Pathogenic
rs757536818	R376C	dbSNP	-3.65	Deleterious	not found
rs761885185	R379C	dbSNP	-2.648	Deleterious	Uncertain significance
rs765502786	R379H	dbSNP	-1.476	Neutral	Uncertain significance
rs1173679499	R393Q	dbSNP	-2.402	Neutral	not found
rs1282887680	R408C	dbSNP	-7.064	Deleterious	not found
rs751698974	R408H	dbSNP	-4.461	Deleterious	not found
rs886039443	F552C	dbSNP	-2.7	Deleterious	Likely pathogenic
rs121908843	C561G	dbSNP	-3.819	Deleterious	Likely pathogenic
rs774221257	L562R	dbSNP	-2.328	Neutral	not found
rs1172845743	Y574C	dbSNP	-2.828	Deleterious	not found
rs774550896	R647H	dbSNP	-2.062	Neutral	not found
rs764601563	R655Q	dbSNP	-1.246	Neutral	not found

Table S3. Regulome DB results of non-coding SNPs

dbSNP IDs	Regulome DB Rank	Regulome DB Score	Type	Position in respect to TP63
rs62290004	2a	0.67948	Intron Variant	Intron 1-2
rs6774934	2a	1	Intron Variant	Intron 1-2
rs11708278	2b	0.68277	Intron Variant	Intron 3-4
rs1913721	2b	0.43292	Intron Variant	Intron 4-5
rs1913722, rs57898901	2b	0.62301	Intron Variant	Intron 4-5
rs4488809	2b	0.57802	Intron Variant	Intron 1-2
rs4687090	2b	0.43292	Intron Variant	Intron 1-2
rs55803942	2b	0.46415	Intron Variant	Intron 3-4
rs56104635	2b	0.70883	Intron Variant	Intron 4-5
rs6444404	2b	0.50526	Intron Variant	Intron 4-5
rs6794898	2b	0.67017	Intron Variant	Intron 1-2
rs6797174	2b	0.93104	Intron Variant	Intron 1-2
rs79155799	2b	0.55744	Intron Variant	Intron 1-2
rs79659066	2b	0.69579	Intron Variant	Intron 3-4
rs9830137	2b	0.70883	Intron Variant	Intron 3-4
rs9847745	2b	0.63796	Intron Variant	Intron 1-2
rs10049472	2c	0.49417	Intron Variant	Intron 1-2
rs4687085	2c	0.42417	Intron Variant	Intron 1-2
rs6777728	2c	0.9	Intron Variant	Intron 4-5
rs28673064	3a	0.97433	5 prime UTR variant	5 prime UTR
rs78233713	7	0.18412	3 prime UTR variant	3 prime UTR
rs73199799	7	0.18412	3 prime UTR variant	3 prime UTR

Table S4. miRNA binding site prediction of noncoding SNPs in TP63 protein through PolymiRTS

Location	dbSNP ID	Variant	Wobble	Ancestral	Allele	miR ID	Conservation	miRSite	Function	Exp	context+
189612062	rs142981128	SNP	Y	G	G	hsa-miR-22-3p	16	accaccGGCAGCT	D	N	-0.161
						hsa-miR-138-5p	14	acCACCAGCAgct	C	N	-0.35
						hsa-miR-3692-5p	14	accaCCAGCAGct	C	N	-0.186
						hsa-miR-4456	13	aCCACCAGcagct	C	N	-0.192
						hsa-miR-4722-5p	14	tctcatCTCCTGC	O	N	-0.168
189612196	rs140149400	SNP	Y	A	A	hsa-miR-1273f	19	cagaCCATCTCtt	D	N	-0.149
						hsa-miR-4527	19	CAGACCAtctctt	D	N	-0.131
						hsa-miR-6503-5p	19	CAGACCAtctctt	D	N	-0.131
						hsa-miR-6753-3p	19	CAGACCAtctctt	D	N	-0.119
						hsa-miR-7107-3p	19	CAGACCAtctctt	D	N	-0.116
					G	hsa-miR-409-5p	12	catatcGGTAACC	C	N	-0.073
189613717	rs36099321	SNP	N	C	C	hsa-miR-184	10	gtttcCCGTCCAt	D	N	-0.135
						hsa-miR-4804-5p	10	gtttcCCGTCCAt	D	N	-0.122
						hsa-miR-4520a-3p	10	gtttcCTGTCCAt	C	N	-0.011
					C	hsa-miR-636	13	tggtaaCAAGCAC	C	N	-0.166
189614414	rs36064124	SNP	N	C	C	hsa-miR-6892-5p	13	ctgctTCCCTTAc	D	N	-0.119
189614507	rs35861864	SNP	Y	G	G	hsa-miR-101-3p	10	tGTACTGTgtctc	D	N	-0.086

Table S5. PolymiRTS Results of noncoding SNPs for disease association

Disease/Trait	PubMedID	MarkerID	*p*_Value
Brain imaging	20100581	rs7610017	NS
Lung cancer	23143601	rs4488809	$4x10^{-9}$
Lung cancer	21725308	rs4488809	$7x10^{-26}$
Lung adenocarcinoma	22797724	rs10937405	$7x10^{-17}$
Lung adenocarcinoma	20871597	rs10937405	$7x10^{-12}$
Acute lymphoblastic leukemia (childhood)	22076464	rs17505102	$9x10^{-9}$
Acute lymphoblastic leukemia (childhood)	22076464	rs17505102	$2x10^{-8}$
Bladder cancer	20972438	rs710521	$2x10^{-10}$
Urinary bladder cancer	20348956	rs710521	$6x10^{-8}$
Urinary bladder cancer	18794855	rs710521	$1x10^{-7}$

T**able S6**: RMSD, Rg, SASA values from MD simulations.

Attributes	Wildtype R319			Mutant319			WildtypeG349			MutantG349			Wild type			Mutant		
	**RMSD**	**Rg**	**SASA**	**RMSD**	**Rg**	**SASA**	**RMSD**	**Rg**	**SASA**	**RMSD**	**Rg**	**SASA**	**RMSD**	**Rg**	**SASA**	**RMSD**	**Rg**	**SASA**
Mean	12.0	19.2	5295.7	13.093	24.6	5450.3	12.0	20.9	5425.6	11.0	20.9	5354.8	8.4	9.7	19.4	17.6	5109.9	5030.1
Min	0.52	13.7	4795.1	0.54	18.5	4880.1	0.68	15.76	4711.9	0.60	16.5	4569.5	0.4	0.40	15.8	13.5	4706.9	4454.0
Max	18.8	28.4	5853.755	19.1	28.7	6060.6	17.6	27.63	6267.8	18.2	29.1	6215.6	24.0	20.3	27.5	24.9	6120.1	5717.6
